# Refractory and progressively worsening nasal obstruction: case report of nasal osteoblastoma and literature review

**DOI:** 10.3389/fonc.2023.1168777

**Published:** 2023-07-14

**Authors:** Caishan Fang, Ruizhi Wang, Min Zhou, Tengyu Chen, Qinxiu Zhang, Yan Ruan, Chunqiao Li

**Affiliations:** ^1^ Hospital of Chengdu University of Traditional Chinese Medicine, Chengdu University of Traditional Chinese Medicine, Chengdu, China; ^2^ The First Clinical Medical College, Guangzhou University of Chinese Medicine, Guangzhou, China; ^3^ Department of Allergy, Third Affiliated Hospital of Sun Yat-Sen University, Guangzhou, China; ^4^ Department of Otolaryngology, Fifth Affiliated Hospital of Sun Yat-Sen University, Zhuhai, China; ^5^ Department of Otolaryngology, First Affiliated Hospital of Guangzhou University of Chinese Medicine, Guangzhou, China

**Keywords:** osteoblastoma, nasal obstruction, osteoid osteoma, fibrous dysplasia of bone, osteoblastic fibroma

## Abstract

Nasal osteoblastoma (OB) is a rare and locally aggressive osteogenic tumor that has rarely been reported, and there is a lack of effective evidence data for its diagnosis and treatment. In this study, we report a 31-year-old female patient who presented with nasal congestion and associated progressive painless swelling of the left maxillofacial region. A preoperative computed tomography (CT) examination of the paranasal sinuses was performed, and based on the imaging presentation, the surgeon was unable to differentiate between OB, osteoid osteoma (OO), fibrous dysplasia of bone (FDB) and osteoblastic fibroma (OF). After excluding contraindications to surgery, the patient underwent nasal endoscopic excision of the left nasal mass, which was found to be gravel-like and difficult to remove cleanly during the operation. The mass was brittle and bled easily, resulting in inadequate exposure of the operative field, prolonged operation time, and substantial intraoperative blood loss. This indicates that definite preoperative diagnosis (biopsy of deeper parts of the mass is recommended) and appropriate preoperative preparations (e.g., preoperative angiography and embolization, adequate blood preparation) are very important. The intraoperative frozen and postoperative pathological results clearly identified the tumor as OB. No local recurrence of the tumor was observed at the 11-month postoperative follow-up.

## Introduction

Osteoblastoma (OB) is a locally aggressive osteogenic tumor that is of intermediate malignancy between benign and malignant (1). OB is a rare clinical disease, accounting for less than 1% of primary bone tumors and occurring predominantly in patients 10-20 years old. OB can occur in multiple parts of the body, most commonly in the spine, followed by the long bones of the limbs, and it occurs in approximately 18-20% of the craniofacial bones (1, 2). Among the craniofacial bones, OB is commonly found in the maxilla, frontal bone and mandible, with localized pain and swelling as the initial symptoms (3–5). In contrast, when OB occurs in the nasal cavity or sinuses, the tumor has clear borders within the structures of the nasal cavity and sinuses, and it usually grows in an expansile manner, easily remodeling without destroying the adjacent bones (4, 6). Because OB growth in the nasal cavity or sinuses is very seldom observed, relevant reports are rare, which makes OB in the nasal cavity or sinuses challenging to diagnose and treat. Herein, we report a case of OB that occurred in the left nasal region, focusing on the clinical diagnostic points and surgical approach, with the aim of improving the diagnosis and treatment of OB in clinical practice.

## Case report

This case report presents a 31-year-old woman who was admitted to the hospital with “recurrent nasal congestion for 3 years, aggravated with left maxillofacial swelling for 1 year”. The patient had recurrent nasal congestion for the past 3 years, especially in the left nasal cavity. Unfortunately, she exhibited painless progressive swelling of the left maxillofacial region for the past 1 year, and the symptoms of nasal congestion on the left side continued to worsen without other symptoms, such as nasal itching, sneezing or runny nose. The patient had a history of chronic rhinitis and had undergone bilateral inferior turbinate plasma ablation for “nasal congestion” in the hospital. At admission, the main symptoms and signs of the patient were obvious nasal congestion on the left side and progressive swelling of the left maxillofacial region without pain.

**Figure 1:**
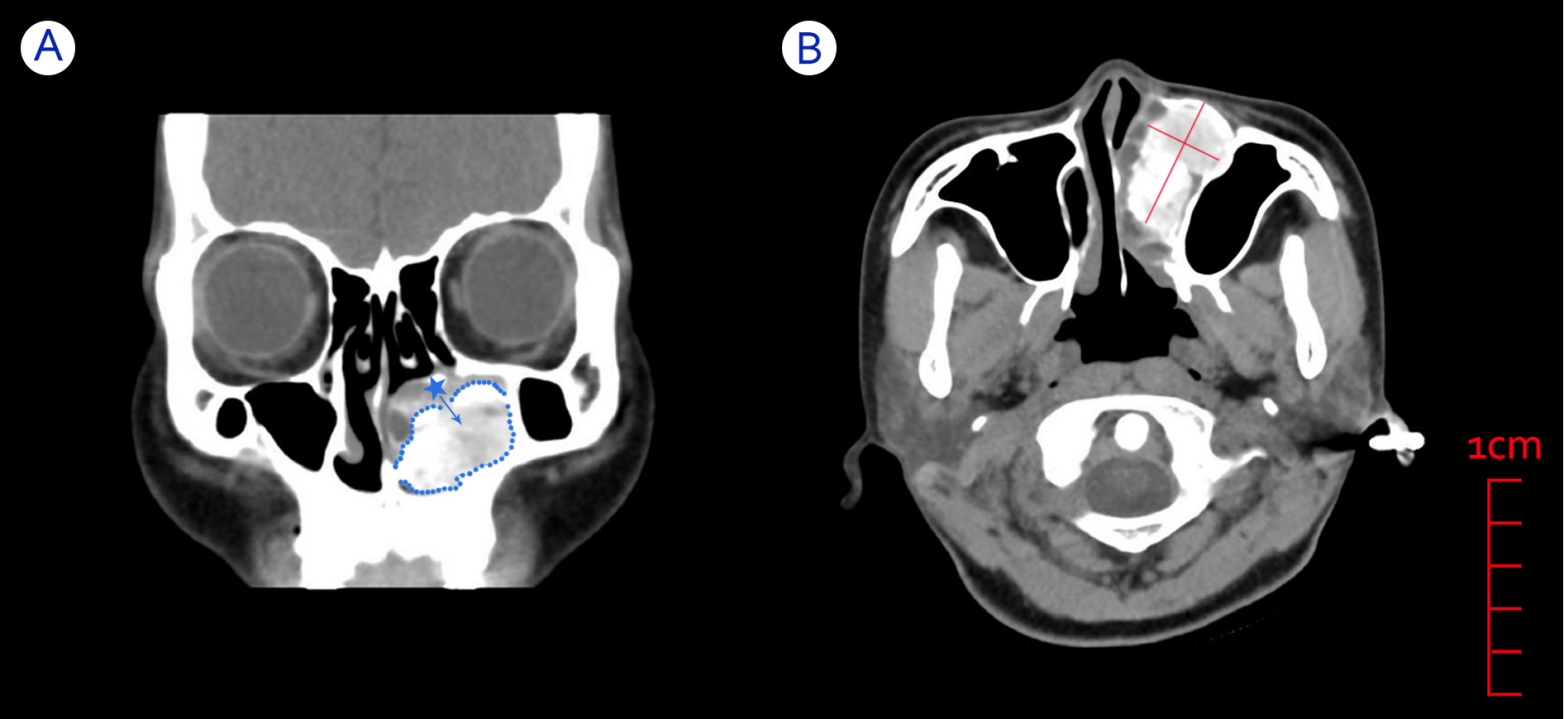
CT images of a patient with an OB in the left nasal cavity. **(A)** Coronal CT showing swelling blocking the left nasal cavity and originating from the inferior turbinate bone, with clearly defined bone between the mass and the surrounding sinus area. Blue dotted line: boundary of the mass. Blue asterisk: left inferior turbinate. Blue arrow: possible growth direction of the mass. **(B)** Axial CT showing a large and well-circumscribed mass compressing adjacent structures such as the left inferior turbinate and maxillary sinus.

After admission to the hospital, the patient underwent relevant examinations. Electronic nasal endoscopy was not performed because the swelling was found to completely block the left nasal cavity during anterior rhinoscopy. Subsequently, a CT scan of the paranasal sinuses was performed (see **Figure 1**) and showed a localized area of dilated low-density bone destruction in the left maxilla near the maxillary sinus and nasal tract, with a size of approximately 5.1 cm * 3.1 cm * 2.4 cm. The boundary of the lesion was clear and suggested a thin shell of calcification. Large flakes of calcification and ossification were observed in the lesion. The lesion obstructed the left middle and lower nasal passages and invaded the left inferior turbinate and the left nasolacrimal duct, surrounded by the adjacent maxillary sinus with localized bone resorption and thinning of the medial wall of the left maxillary sinus, the nasal septum, and the left side of the superior alveolus. The periosteal reaction was not obvious, and there was no surrounding soft tissue mass. The imaging physician diagnosed the abnormal density of the left maxillary sinus as potentially an OO or tumor-like lesion, FDB, or OF, and further clarification of the diagnosis with clinical examination or MRI was recommended. Thus, it was difficult for the imaging physician to identify this lesion on CT alone.

Sequential CT imaging showed that the mass was growing outward and compressing the medial wall of the left maxillary sinus, the nasal septum, and the superior alveolus. Since the border between the left inferior turbinate bone and the high-density shadow of the mass was unclear, we speculated that the mass might originate from the inferior turbinate bone. Combined with the patient’s clinical symptoms and signs, we preoperatively considered that the patient’s left nasal mass might be OB or OF. Therefore, the patient was scheduled for elective resection of the left nasal mass.

**Figure 2:**
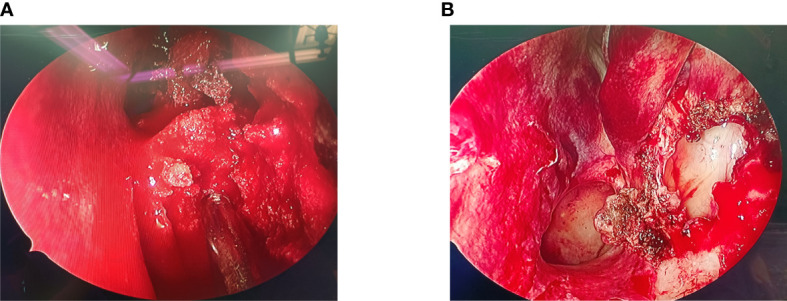
**(A)** Intraoperative nasal endoscopy revealed a soft mass with gravel-like changes in the left nasal cavity, which bled heavily when touched, blurring the surgical field. **(B)** After the mass was removed, the enlarged maxillary sinus opening was visible under the nasal endoscope, and the middle turbinate and the medial wall of the maxillary sinus were not invaded by the mass.

**Figure 3:**
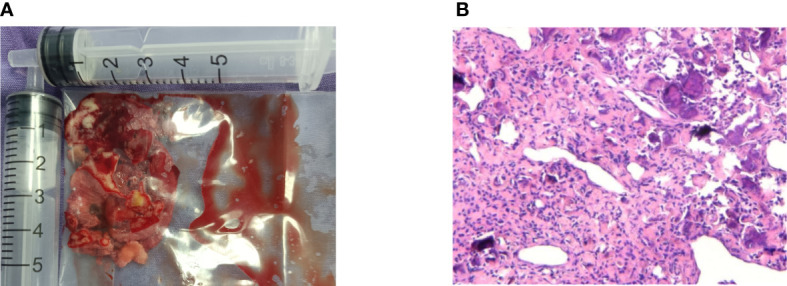
Image of the left nasal mass gross specimen and tissue. **(A)** Total volume of tumor removed during surgery. **(B)** High magnification view of histologic image demonstrating massive irregularly calcified bone-like tissues, proliferated fibers and blood vessels, as well as osteoblasts around the trabecular bone (hematoxylin-eosin, original magnification 100×).

During the operation, we found that the mass (**Figure 2**) was soft and “gravel-like”, and it was difficult to completely remove and clean up. In addition, due to the large amount of bleeding during removal of the mass, the surgical field could not be exposed clearly, resulting in a prolonged operative time (the total operation time was 155 minutes), which in turn further increased the intraoperative bleeding (the final volume of bleeding was approximately 1000 mL). Laboratory tests indicated that the patient’s preoperative hemoglobin level was 127 g/L, while the postoperative hemoglobin level was only 72 g/L. The postoperative pathological findings (**Figure 3**) showed that the mass consisted of a large amount of irregularly calcified bone-like tissue, proliferating fibers and blood vessels, and osteoblasts were seen around the bone trabeculae. Based on the patient’s clinical presentation and imaging findings, the mass was consistent with OB-like changes in the left nasal cavity. The patient was discharged from the hospital and underwent follow-up for 11 months, over which time the nasal congestion and facial swelling improved significantly. Follow-up nasal endoscopy showed that the patient’s left nasal mucosa recovered well, and CT of the paranasal sinuses did not show any recurrence of the mass.

## Differential diagnosis

OB must be distinguished from FDB, OF and OO, as they have very similar imaging presentations (1), especially when the mass occurs on the maxillofacial bone. There is no major difference between their density presentation on CT, and their clinical manifestations and characteristics, growth patterns and clarity of the lesion boundary are the main points of differentiation (1, 7), which are worthy of attention in the process of clinical diagnosis.

FDB: Patients often present with a painless bony bulge; it is only when the mass enlarges and compresses the surrounding tissues that it causes the corresponding clinical symptoms, such as nasal congestion when the nasal sinuses are involved (8, 9). FDB is characterized by hypertrophy of the sinus wall on CT, involving multiple bones, and the lesions are often diffuse and poorly demarcated from the surrounding normal tissue, typically showing “ground glass-like” changes. The lesion usually contains a soft tissue density shadow above the muscle and a speckled, flocculent calcium-like high-density shadow, which corresponds to hyperplastic fibrous tissue and FDB. In addition, FDB can be malignant in the clinic (8, 10, 11).

OF: OF is also called cemento-ossifying fibroma. The disease tends to occur in adolescents. Based on the imaging features, OF occurring in childhood or adolescence can be subdivided into juvenile trabecular OF and juvenile psammomatoid OF (12). Clinically, OF mainly presents as painless and progressive facial swelling. The lesion is well defined and appears as a “ground glass-like” slightly high- or high-density shadow on the CT bone window, with internal cystic cavity formation sometimes accompanied by denser foci of punctate calcification; some of the lesions may have an “eggshell-like” bone shell formation around the periphery (13), with an annular or curved low-density shadow on the inner side. The lesions occur in the bone cortex, showing osteolytic bone destruction, often with uneven density, accompanied by thinning of the bone cortex with clearer borders than FDB, narrowing of the medullary cavity, and no soft tissue masses. If the lesion is predominantly fibrous tissue, it appears as a cystic lesion on X-ray and CT with a round or irregularly shaped bone destruction area, with single or multiple areas of bone swelling, mostly with clear osteosclerotic margins, not involving the epiphysis, and generally without periosteal reaction. If the lesion is predominantly bone tissue, the lesion appears as a sclerotic lesion on X-ray and CT with a high density similar to normal bone, and in some cases even a uniform dense bone mass (12–14).

OO: OO and OB are both benign bone-forming tumors, but OO is not locally aggressive and generally has a low probability of malignancy (15, 16). In terms of clinical signs and symptoms, both present with pain, local tissue swelling and tenderness. However, the pain in OO is typically worse and more frequent at night and is relieved with the use of nonsteroidal anti-inflammatory drugs (NSAIDs). In contrast, the pain symptoms of OB are milder than those of OO and do not worsen at night, but the pain and local masses develop progressively (15), and the use of NSAIDs does not relieve the pain symptoms. The disease has a long course, and the masses tend to develop gradually and become larger. OO appears as an isolated bony mass with well-defined borders on CT, often with central mineralization without soft tissue density and with varying degrees of sclerosis of the surrounding tissue, and the volume of the lesion is usually less than 2 cm in diameter (17). The typical CT presentation of OB also shows mineralization in the central area of the lesion, with expansive bone remodeling, peripheral reactive sclerosis and a thin marginal shell (15, 18, 19). The most important difference between OO and OB in CT imaging is whether the bony marginal area of the tumor is invasive to other surrounding tissues. However, in clinical practice, not all patients’ CT images are clearly distinguishable, so the differential diagnosis of OO and OB is difficult when the clinical features and imaging manifestations of the mass are not typical.

## Discussion

The clinical presentation of OB is nonspecific. Its preoperative diagnosis mainly relies on imaging and pathological examinations, with the main imaging examination methods including CT and MRI. CT imaging of OB has shown that bone shells of uneven thickness can form around the tumor, and calcification of different shapes can be seen inside the tumor. The MRI findings of osteoblastoma are not specific compared to those of other bone tumors. On T1-weighted imaging, osteoblastoma lesions show low- to moderate-intensity signals. The presentation on T2-weighted imaging is more varied, with low-, medium-, or high-intensity signals in different cases. Calcification shadows inside the lesion may show different degrees of hypointensity on T2-weighted imaging, and osteosclerosis around the lesion is characterized by annular low-intensity shadows on T2 imaging (20, 21). Compared with CT, MRI has advantages in showing the tumor scope, soft tissue masses, peripheral edema, and relationship with surrounding tissues; however, it is inferior to CT in showing scattered areas of spotty calcification inside the tumor, degree of damage to cortical bone, and sclerotic edges around the lesion (22). CT offers very high resolution and can directly display bone destruction and bone shell morphology. Additionally, CT can reveal fine calcification and ossification inside the lesion and the sclerotic edge of the bone cortex to determine whether the bone cortex is interrupted. Hence, CT is still the most effective imaging method for OB (15, 23). Preoperative CT can be used to accurately display and locate the tumor and determine the degree of bone involvement, which is of great value in preoperative staging and surgical planning (24–26).

However, the paranasal sinus CT results of this patient showed that the mass had a mixed-density appearance, with mainly high-density shadows, which did not distinguish it from OO, OF, or FDB, as previously described. Although the most effective treatment for these diseases is complete surgical excision of the mass (10, 13, 15), the preoperative preparation and intraoperative techniques are different. If the doctor is still unable to make a definite diagnosis before surgery using a combination of clinical symptoms, signs and imaging findings, then preoperative pathological examination can be considered. In six cases of sinus OB reported by Ning Ma et al. (27), none of which could be diagnosed preoperatively, pathological tissues were routinely taken for preoperative biopsy, but the small amount of pathological tissue sampled and the shallow location did not provide sufficient appropriate pathological tissues for the pathology department. This suggests that special attention should be given to obtaining deeper tissue samples during preoperative biopsy of OBs.

Nasal OB is a rare disease with few clinical reports, and there is a lack of evidence and consensus on effective treatment. According to the available literature, surgery remains the most effective treatment for OB (15). Since the disease has locally aggressive behavior and can cause damage to the surrounding tissue and bone, the choice of the appropriate surgical approach depends largely on the location and extent of tumor invasion. Intralesional curettage and en bloc resection are the most common surgical treatments for this disease (15, 18). Compared with other benign tumors, such as OO, OB has a relatively high recurrence rate, so surgical en bloc resection can effectively reduce tumor recurrence (15, 28). When the mass is confined to the nasal cavity or sinuses, nasal endoscopic surgery is the best surgical approach. However, if the mass is large or invades surrounding tissues, making surgical excision with nasal endoscopy difficult, other surgical approaches, such as the open approach, should be considered.

In this case report, the patient’s preoperative diagnosis was a left-sided nasal OO. Since it was a localized, isolated lesion with clear borders and a well-defined hard border surrounding it, the operator believed that complete resection of the lesion would be possible along the bony border during surgical excision and thus performed only routine preoperative preparation. However, during surgical resection, the mass, although of bony origin, was found to be soft, gravel-like, brittle, and prone to bleeding. Intraoperative bleeding caused an unclear field, which made the operation more difficult and significantly lengthened the operation, further increasing intraoperative bleeding and other potential risks, such as those due to surgical anesthesia. Postoperative blood analysis revealed that the patient’s hemoglobin volume was reduced by more than half compared to the preoperative volume, further confirming the high intraoperative blood loss of this patient.

In summary, this case suggests that OB tumors may have abundant blood flow. Preoperative blood preparation and intraoperative bleeding control are important. As a consequence, preoperative prophylactic angiography and embolization as well as adequate blood preparation should be comprehensively considered when dealing with patients of the same type. During the operation, autologous blood transfusion can be performed to effectively control the patient’s blood pressure to further reduce the risk of intraoperative bleeding.

## Conclusion

Based on this case review, we can draw the following conclusions. First, for progressive nasal congestion for which conventional treatment in the clinic is ineffective, it is necessary to improve in-depth examinations, such as CT scans of the paranasal sinuses, to avoid missing a diagnosis of related tumor-like diseases. Second, for nasal bone tumor diseases with an unclear diagnosis after preoperative CT examination, it is recommended to improve pathological examinations before surgery and try to collect deeper tissue samples during biopsy to clarify the diagnosis. Finally, if the preoperative diagnosis of nasal OB is definitive, preoperative angiography and embolization and adequate blood preparation should be considered sufficiently. During the operation, autologous blood transfusion and active blood pressure control can be performed to further reduce the risk of intraoperative bleeding.

## Data availability statement

The original contributions presented in the study are included in the article/supplementary material. Further inquiries can be directed to the corresponding authors.

## Ethics statement

The studies involving human participants were reviewed and approved by the Ethics Committee of The First Affiliated Hospital of Guangzhou University of Chinese Medicine (File No. ZYYECK [2022]041). The patients/participants provided their written informed consent to participate in this study. Written informed consent was obtained from the participant/patient(s) for the publication of this case report.

## Author contributions

CL and CF participated in the clinical diagnosis and treatment of this reported case. CF and RW wrote the manuscript, and QZ helped check the text for grammatical errors. MZ and TC collected the clinical data. YR provided funding support. All authors contributed to the article and approved the submitted version.

## References

[1] Izzo A Zugaro L Fascetti E Bruno F Zoccali C Arrigoni F. Management of osteoblastoma and giant osteoid osteoma with percutaneous thermoablation techniques. J Clin Med (2021) 10(24):5717. doi: 10.3390/jcm10245717 34945013 PMC8709302

[2] Wu M Xu K Xie Y Yan F Deng Z Lei J. Diagnostic and management options of osteoblastoma in the spine. Med Sci Monit (2019) 25:1362–72. doi: 10.12659/MSM.913666 PMC6391855 30785872

[3] Limaiem F Byerly DW Singh R. Osteoblastoma. In: StatPearls. Treasure Island (FL: StatPearls Publishing (2022). 30725639

[4] Chen KT Weinberg RA Simpson PR Tschang TP. Osteoblastoma of the nasal cavity. J Laryngol Otol (1993) 107(8):737–9. doi: 10.1017/s0022215100124296 8409731

[5] Young E Dabrowski M Brelsford K. Osteoblastoma of the nasal septum. J Laryngol Otol (2011) 125(10):1062–6. doi: 10.1017/S0022215111001708 21806856

[6] Kiyohara H Sawatsubashi M Matsumoto N Komune S. Benign osteoblastoma of the ethmoid sinus. Auris Nasus Larynx (2013) 40(3):338–41. doi: 10.1016/j.anl.2012.07.003 22867522

[7] Fu YS Perzin KH. Non-epithelial tumors of the nasal cavity, paranasal sinuses, and nasopharynx. a clinicopathologic study. II. osseous and fibro-osseous lesions, including osteoma, fibrous dysplasia, ossifying fibroma, osteoblastoma, giant cell tumor, and osteosarcoma. Cancer (1974) 33(5):1289–305. doi: 10.1002/1097-0142(197405)33:5<1289::aid-cncr2820330514>3.0.co;2-p 4207295

[8] Chattopadhyay A Jain S Sharma A. Craniofacial fibrous dysplasia. J Clin Rheumatol (2020) 26(7):e214. doi: 10.1097/RHU.0000000000001082 31246705

[9] Tafti D Cecava ND. Fibrous dysplasia. In: StatPearls. Treasure Island (FL: StatPearls Publishing (2022). 30422542

[10] Burke AB Collins MT Boyce AM. Fibrous dysplasia of bone: craniofacial and dental implications. Oral Dis (2017) 23(6):697–708. doi: 10.1111/odi.12563 27493082 PMC5292317

[11] Boyce AM Collins MT. Fibrous Dysplasia/McCune-albright syndrome: a rare, mosaic disease of gα s activation. Endocr Rev (2020) 41(2):345–70. doi: 10.1210/endrev/bnz011 PMC7127130 31673695

[12] Chrcanovic BR Gomez RS. Juvenile ossifying fibroma of the jaws and paranasal sinuses: a systematic review of the cases reported in the literature. Int J Oral Maxillofac Surg (2020) 49(1):28–37. doi: 10.1016/j.ijom.2019.06.029 31285096

[13] Manes RP Ryan MW Batra PS Mendelsohn D Fang YV Marple BF. Ossifying fibroma of the nose and paranasal sinuses. Int Forum Allergy Rhinol (2013) 3(2):161–8. doi: 10.1002/alr.21067 22736440

[14] Pirana S Zerati F Voegels R Maia R. Psammomatoid ossifying fibroma. Rhinology (2003) 41(4):250–2. 14750354

[15] Atesok KI Alman BA Schemitsch EH Peyser A Mankin H. Osteoid osteoma and osteoblastoma. J Am Acad Orthop Surg (2011) 19(11):678–89. doi: 10.5435/00124635-201111000-00004 22052644

[16] Lam SW Cleven AHG Kroon HM Briaire-de Bruijn IH Szuhai K Bovée JVMG. Utility of FOS as diagnostic marker for osteoid osteoma and osteoblastoma. Virchows Arch (2020) 476(3):455–63. doi: 10.1007/s00428-019-02684-9 PMC7085481 31768625

[17] Cobianchi Bellisari F Palumbo P Masciocchi C Zoccali C Barile A Arrigoni F. Needleless ablation of osteoid osteoma and osteoblastoma: the emergent role of MRgFUS. J Clin Med (2021) 11(1):128. doi: 10.3390/jcm11010128 35011867 PMC8745067

[18] Villalobos CE Rybak LD Steiner GC Wittig JC. Osteoblastoma of the sternum–case report and review of the literature. Bull NYU Hosp Jt Dis (2010) 68(1):55–9. 20345366

[19] Ellingsen T Nalley A Oda D Dodson TB Lee PP. Osteoblastoma and osteoid osteoma of the mandible: review of the literature and report of two cases. Case Rep Dent (2022) 2022:7623855. doi: 10.1155/2022/7623855 35300290 PMC8923807

[20] Zhang GF Liu Y. Osteoblastoma of left middle cranial fossa: case report. Chin J Med Imaging Technol (2019) 35(20):1922. doi: 10.13929/j.1003-3289.201901084

[21] Wang C Jiang L Liu ZJ. Basic and clinical research advancement of spinal osteoblastoma. Chin J Spine Spinal Cord (2013) 23(12):1122.

[22] Koc K Ilik MK. Surgical management of an osteoblastoma involving the entire C2 vertebra and a review of literature. Eur Spine J (2016) 25(Suppl 1):220–3. doi: 10.1007/s00586-016-4445-0 26879919

[23] Papaioannou G Sebire NJ McHugh K. Imaging of the unusual pediatric ‘blastomas’. Cancer Imaging (2009) 9(1):1–11. doi: 10.1102/1470-7330.2009.0001 19237343 PMC2651735

[24] Dickman CA Fehlings MG Gokaslan ZL. Spinal cord and spinal column tumors: principles and practice. New York: Thieme (2005), 33–4.

[25] Woertler K. Benign bone tumors and tumor-like lesions: value of cross-sectional imaging. Eur Radiol (2003) 13(8):1820–35. doi: 10.1007/s00330-003-1902-z 12700923

[26] Zileli M Cagli S Basdemir G Ersahin Y. Osteoid osteomas and osteoblastomas of the spine. Neurosurgical Focus (2003) 15(5):1–7. doi: 10.3171/foc.2003.15.5.5 15323462

[27] Ma N Weng D. Osteoblastoma of sinuses: report of 6 cases. J Clin Otorhinolaryngol (2000) 14(11):515–6.

[28] Berry M Mankin H Gebhardt M Rosenberg A Hornicek F. Osteoblastoma: a 30-year study of 99 cases. J Surg Oncol (2008) 98(3):179–83. doi: 10.1002/jso.21105 18561158

